# The role of insulin resistance in experimental diabetic retinopathy—Genetic and molecular aspects

**DOI:** 10.1371/journal.pone.0178658

**Published:** 2017-06-02

**Authors:** Patrick Järgen, Axel Dietrich, Andreas W. Herling, Hans-Peter Hammes, Paulus Wohlfart

**Affiliations:** 1 5^th^ Medical Department, Universitätsmedizin Mannheim (UMM), University of Heidelberg, Mannheim, Germany; 2 Sanofi Aventis Deutschland GmbH, R&D Lead Generation Candidate Realization, Structure, Design and Informatics, Industriepark Höchst, Frankfurt, Germany; 3 Sanofi Aventis Deutschland GmbH, TA Diabetes R&D, Industriepark Höchst, Frankfurt, Germany; University of Florida, UNITED STATES

## Abstract

**Background:**

Diabetic retinopathy is characterized by defects in the retinal neurovascular unit. The underlying mechanisms of impairment–including reactive intermediates and growth-factor dependent signalling pathways and their possible interplay are incompletely understood. This study aims to assess the relative role of hyperglycemia and hyperinsulinemia alone or in combination on the gene expression patterning in the retina of animal models of diabetes.

**Material and methods:**

As insulinopenic, hyperglycemic model reflecting type 1 diabetes, male STZ-Wistar rats (60mg/kg BW; i.p. injection at life age week 7) were used. Male obese ZDF rats (fa/fa) were used as type-2 diabetes model characterized by persisting hyperglycemia and transient hyperinsulinemia. Male obese ZF rats (fa/fa) were used reflecting euglycemia and severe insulin resistance. All groups were kept till an age of 20 weeks on respective conditions together with appropriate age-matched controls. Unbiased gene expression analysis was performed per group using Affymetrix gene arrays. Bioinformatics analysis included analysis for clustering and differential gene expression, and pathway and upstream activator analysis. Gene expression differences were confirmed by microfluidic card PCR technology.

**Results:**

The most complex genetic regulation in the retina was observed in ZDF rats with a strong overlap to STZ-Wistar rats. Surprisingly, systemic insulin resistance alone in ZF rats without concomitant hyperglycemia did not induce any significant change in retinal gene expression pattern. Pathway analysis indicate an overlap between ZDF rats and STZ-treated rats in pathways like complement system activation, acute phase response signalling, and oncostatin-M signalling. Major array gene expression changes could be confirmed by subsequent PCR. An analysis of upstream transcriptional regulators revealed interferon-γ, interleukin-6 and oncostatin-M in STZ and ZDF rats. CONCLUSIONS: Systemic hyperinsulinaemia without hyperglycemia does not result in significant gene expression changes in retina. In contrast, persistent systemic hyperglycemia boosts much stronger expression changes with a limited number of known and new key regulators.

## Introduction

The global incidence of diabetes is set to rise dramatically worldwide. Approximately one tenth of diagnosed patients suffer from type-1 diabetes (T1D), which arises from an autoimmune destruction of beta cells in the endocrine pancreas causing severe insulin deficiency. More than 90% of patients however display a type-2 diabetes phenotype (T2D), which is characterized in a first step by impaired insulin action in peripheral tissues, and initially high compensating endogenous levels of insulin. Due to exhaustion and failure of beta cells, impaired insulin production is observed in later stages. Patients with long-duration diabetes develop cardiovascular complications resulting in highly increased mortality and complications which affect the kidneys, eyes and peripheral nerves associated with high morbidity. The mortality and morbidity caused by diabetes threatens healthcare systems worldwide.

Among diabetic complications, damage in the eye, diabetic retinopathy, is a common microvascular complication of diabetes. It includes diabetic macular edema, a leading global cause of vision-loss [1]. Among diabetes patients worldwide, approximately one third have signs of diabetic retinopathy and of these, a further one third is vision-threatening [2]. Currently established therapeutic options only partially suffice to slow disease progression. Intra-vitreal injections of scavengers of vascular endothelial growth factor and antiangiogenic steroid therapies fail in half of the patients. Being destructive to large parts of the retina, laser photocoagulation remains standard therapy for advanced stages of diabetic retinopathy underlining a gap in much more efficient therapies in earlier disease stages [1].

Important risk factors for diabetic retinopathy are elevated long diabetes duration and systemic glucose levels. Normalization of blood glucose levels would be the ultimate goal, but is difficult to achieve despite increasing numbers of established glucose lowering therapies. Hyperglycemia is not the sole cause of retinal injury. Other clinical metabolic factors are altered in diabetes, and elevated blood pressure is certainly of importance. Insulin therapy in T1D and hyperinsulinemia in T2D have been discussed as a cause of both, onset and progression of retinopathy [3].

Diabetic retinopathy is characterized by a bundle of patho-mechanisms including capillary degeneration, altered permeability, and, at a late stage, excess formation of new blood vessels [4]. Beyond such vascular changes, changes in the neurovascular unit may be of similar importance [1] [5]. Models of retinopathy in non-human mammalian species do not cover final neovascularization like in man, maybe due to the shorter lifespan of such species. However, these preclinical models develop similar early diseases, and maybe therefore well suited to identify early disease-initiating mechanisms. We recently performed gene expression analysis in a rodent T2DM model of retinopathy showing that growth factors like fibroblast growth factor 2 (FGF2) and placental growth factor (PGF) may be involved in the disease progression [6]. In this study, we used unbiased gene expression analysis via microarrays to compare retinal gene regulation by hyperglycemia, equivalent to type-1 diabetes, to retinal gene expression in response to systemic hyperinsulinemia in conjunction without and with hyperglycemia to uncover the relative contributions of such conditions to molecular regulations.

## Materials and methods

### Animal study

The animal study was carried out at Sanofi Aventis Deutschland GmbH in Frankfurt, Germany in strict accordance with the terms of the German Animal Protection Law and international animal welfare legislation and rules. Sanofi as institution has an Institutional Animal Care and Use Committee (IACUC) termed AWB (Animal Welfare Body). The specific study protocol (permit number 12–001) was approved by this committee before study start, ensuring that all duties according to European Community regulations 2010/63 and German Animal Welfare Law were fulfilled. All surgery was performed under overdosing isoflurane anesthesia, and all efforts were made to minimize suffering.

Male ZDF rats (ZDF-Lepr^fa^-Crl) were obtained from Charles River Laboratories (Kissleg, Germany). Male ZF rats (HsdOla:ZUCKER-Lepr^fa^) and male Wistar rats (HsdCpb:WU) were from Harlan, UK and Harlan, The Netherlands, respectively. ZDF and ZF rats were obtained in two genotypes, obese (genotype fa/fa) and lean littermates (genotype fa/?). All rats were obtained at a life age of 6 weeks and housed in standard cages under a normal light-dark cycle for additional 14 weeks till a final age of 20 weeks. All animals had free access to food and water. ZF and Wistar rats received a standard chow (Ssniff R/M) and ZDF rats received Purina 5008 chow. A group size of n = 8 were used for all study groups. Wistar rats were rendered type-1 like hyperglycemic and hypoinsulinemic via a single injection of streptocotocin (STZ, 60mg/kg; i.p.) at 7 weeks of age. Obese ZDF rats (fa/fa) develop spontaneously a type-2 diabetes phenotype with persisting hyperglycemia and transient hyperinsulinemia (hyperglycemic, hypoinsulinemic). Obese ZF rats (fa/fa) develop insulin resistance with permanent hyperinsulinemia without concomitant hyperglycemia and no overt diabetes phenotype. Non STZ treated Wistar rats, lean ZDF littermates (fa/?), and lean ZF littermates (fa/?) served as controls. Animals were kept on standardized conditions and at week 20 anaesthetized with an overdose of isoflurane and sacrificed, followed by RNA isolation from selected organs. A minimum of 6 animals per group were used in all subsequent analyses.

### Assessments of metabolism and biomarkers

Body weight, food and water consumption, as well as blood glucose were monitored once weekly. Blood for weekly determination of plasma glucose as well as for serum insulin (once after 7 weeks) measurements was taken from the tail vein. At the end of the study on the day before euthanizing the animals, blood (100μl) was drawn under isoflurane anaesthesia for plasma determination of methylglyoxal. Animals were then starved for 16h to determine fasting blood glucose and insulin levels. Subsequently, animals were anesthetized in isoflurane and euthanized by terminal blood collection from the aorta after laparotomy. Serum was collected to determine free fatty acids, triglyceride, cholesterol, ketone bodies, creatinine, urea and uric acid by standard means. Eyes were enucleated and immediately frozen at −80°C until further analysis.

### Differential gene expression measurements

Isolation of RNA from eyes was performed as described [6]. Briefly, retinal RNA was isolated from individual retinas homogenized in 1ml trizol and 200μl chloroform. Aqueous phase of 500μl was obtained and mixed with 500μl of isopropanol. After centrifugation, RNA pellets were washed trice in 80% Ethanol and finally stored in 30μl of DEPC-water. Gene expression profiling was performed using arrays of RaGene-1_0-st-v1-type from Affymetrix. Biotinylated antisense cRNA was then prepared according to the Affymetrix standard labelling protocol with the GeneChip® WT Plus Reagent Kit and the GeneChip® Hybridization, Wash and Stain Kit (both from Affymetrix, Santa Clara, USA). Afterwards, the hybridization on the chip was performed on a GeneChip Hybridization oven 640, then dyed in the GeneChip Fluidics Station 450 and thereafter scanned with a GeneChip Scanner 3000. All of the equipment used was from the Affymetrix-Company (Affymetrix, High Wycombe, UK).

Deposited microarray data are accessible in Array Express following the access code E-MTAB-5563 and the link given in the reference [7].

### Data processing and statistical analysis

Probe level data (CEL files) were processed using Array Studio (Version 8, Omicsoft Corporation, Research Triangle Park, NC, USA). For each microarray dataset, the probe set intensities were normalized using robust multi-array average (RMA) procedure. Only probe sets with at least two values per condition above a threshold intensity value of 64, 2^6^ or 6 on a log2 scale, were regarded as significant. The goal of this filtering by signal is to remove genes that have signal very close to background level [8] This analysis resulted in remaining 16,480 probe sets. Subsequent analyses were performed on natural log-transformed data to reduce the effect of any potential outliers in the data. Log2 intensities of each probe set from all control samples per group were averaged and subtracted from the disease-model samples to generate the corresponding disease-to-control values. These disease-to-control values are referred as expression values.

An one-way ANOVA was performed to determine which genes were significantly differentially expressed between involved and non-involved groups, and Benjamini-Hochberg false discovery rate (FDR) multiple testing correction was applied [9]. Genes were considered significantly altered if p(FDR) <0.03 and the absolute fold-change in expression was ≥1.5.

### Gene expression confirmation

A confirmatory PCR using micro-fluidic cards in 96-well format was performed as described [6]. A number of 49 genes were selected based on highest fold changes between the groups. Three reference genes served for normalization. After performing the real-time PCR with a maximum of 40 cycles, threshold quantification cycle (Cq) were evaluated for each gene using the manufacturer’s software. Those Cq values were imported and analysed using ArrayStudio. Stability of reference gene expression was assessed. Relative gene expression was then obtained by normalizing to a mean housekeeper value, followed by a clustering and outlier analysis by principal component analysis. Finally, differentially regulated genes were identified based on ANOVA analysis. Genes were considered significantly altered if p(FDR) <0.05.

### Pathway annotation of differential gene expression data

Differentially expressed genes were analysed in a next step by the use two complementary software interaction databases, Ingenuity® Pathway Analysis (IPA®, QIAGEN Redwood City, www.qiagen.com/ingenuity) and Metacore Integrated Pathway analysis (Thomson Reuters, ip-science.thomsonreuters.com). Fisher’s Exact test was used to determine the statistical significance of each biological function assigned to the gene expression data.

### Upstream regulator analysis

Upstream Regulator Analysis in IPA incorporates expression of downstream target genes from the experimental dataset and compiled knowledge of reported relationships between regulators and their known target genes [10]. The tool was used to predict upstream regulators and infer their activation state by calculating a z-score that determines whether gene expression changes for known targets of each regulator. An absolute z-score of below -2 (inhibited) or above +2 (activated) was considered significant.

## Results

### Phenotypic differences in metabolic markers and biomarkers

**Fig 1:**
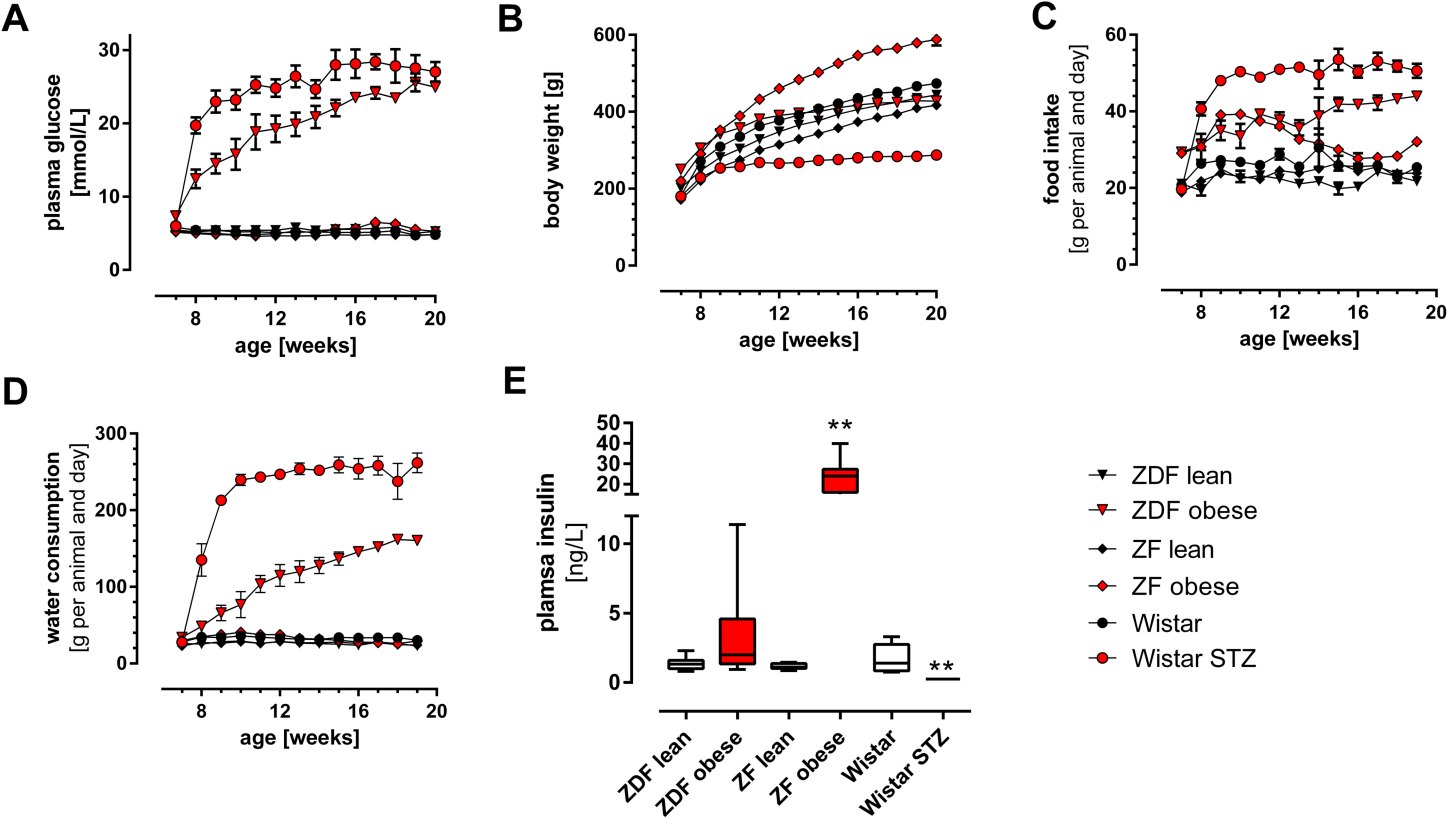
Metabolic parameters in ZDF obese versus ZDF lean rats, ZF obese versus ZF lean rats and Wistar rats without and with single injection of streptozotocin. **A**, Blood glucose monitored weekly in fed animals **B**, Body weight **C,** Food intake, **D,** Water intake; and **E** plasma insulin concentrations in fed animals, Mean ± SEM (n = 8) are shown; *) indicates p<0,05 and **) indicates p<0,01 in unpaired student t-test compared to respective control groups.

Blood glucose, body weight and food and water intake were measured weekly. Beginning at the age of 8 weeks ZDF obese rats displayed increased plasma glucose levels up to 25.6 ± 3.5 mmol/L, and hyperglycemia in STZ-treated Wistar rats increased to similar high levels (28.4 ± 2.8 mmol/L) (Fig 1A). Obese and lean ZF rats as well as Wistar and ZDF lean rats displayed normoglycemic conditions. Measurement of postprandial insulin is shown in Fig 1E. Whereas ZDF obese rats displayed at the end of the study simultaneously hyperglycemia and hyperinsulinemia with Insulin levels of 3.4 ng/L, hyper-glycaemia with hypo-insulinemia was observed in STZ-injected Wistar rats. In ZF obese rats, highly elevated insulin concentrations up to 23.5 ng/L could be measured. Body weight increased most strongly in ZF obese rats (Fig 1B). Body weight after STZ injection in Wistar rats climbed only slightly reaching a low plateau till the end of the study. Control animals and ZF obese consumed 30 ± 4 g/d of water; ZDF obese and Wistar STZ animals showed a strongly increased water intake of 111 ± 43 g/d and 222 ± 67 g/d (Fig 1D).

At the end of the study we determined in all three models a number of metabolic markers and biomarkers characterizing more in-depth the diabetic status (Table 1). Free fatty acids (FFA) and triglycerides were elevated in both, ZDF and ZF obese versus lean animals. Creatinine concentration decreased in all three models, whereas, urea slightly increased in ZDF obese and more strongly in Wistar-STZ rats. Of note, ketone bodies, including hydroxy-butyrate and aceto-acetate were significant higher in obese versus lean ZDF rats, but not in ZF and STZ-treated animals, reflecting hepatic insulin resistance in ZDF only. Plasma methylglyoxal gradually increased in ZDF obese rats and Wistar-STZ. In ZF rats baseline levels were higher compared to the other groups and not different between obese and lean phenotype.

**Table 1:** Plasma biomarkers and metabolites at the end of the study. Shown are mean ± SD values obtained at fasting stage at the end of study.

	ZDF lean	ZDF obese	Wistar	Wistar STZ	ZF lean	ZF obese
**FFA (mmol/L)**	0,53 ± 0,09	0,72 ± 0,08*	0,36 ± 0,07	0,44 ± 0,10	0,53 ± 0,05	0,93 ± 0,14*
**Triglycerides (mmol/L)**	0,58 ± 0,13	7,09 ± 0,45*	0,45 ± 0,11	1,53 ± 0,69*	0,41 ± 0,07	4,44 ± 1,16*
**Cholesterol (mmol/L)**	2,45 ± 0,16	6,56 ± 0,59*	2,08 ± 0,18	2,28 ± 0,40	2,15 ± 0,14	5,03 ± 0,65*
**Creatinine (μmol/L)**	25,75 ± 3,49	14,63 ± 0,74*	26,00 ± 2,98	15,25 ± 2,25*	26,50 ± 1,31	17,13 ± 1,89*
**Urea (mmol/L)**	4,49 ± 0,66	7,61 ± 0,80*	5,50 ± 0,52	23,44 ± 3,77*	5,65 ± 0,56	4,32 ± 0,61*
**Uric Acid (μmol/L)**	48,50 ± 9,24	49,75 ± 13,41	41,75 ± 12,48	43,75 ± 17,83	63,50 ± 13,09	67,38 ± 11,01
**Ketone bodies total (μmol/L)**	1484 ± 366	5571 ± 4128*	1706 ± 274	1050 ± 1331	1668 ± 322	976 ± 411*
**beta hydroxybutyrate (μmol/L)**	1123 ± 288	3601 ± 2600*	1204 ± 244	646 ± 718	1136 ± 272	502 ± 188*
**Acetoacetate (μmol/L)**	362 ± 125	1970 ± 1533*	502 ± 79	404 ± 616	532 ± 101	474 ± 235
**Methylglyoxal (nM)**	1458 ± 222	1854 ± 240*	1797 ± 184	1944 ± 238	1954 ± 237	1955 ± 258

*) denotes p<0.05 vs control animals.

### Gene expression changes in the retina

**Fig 2:**
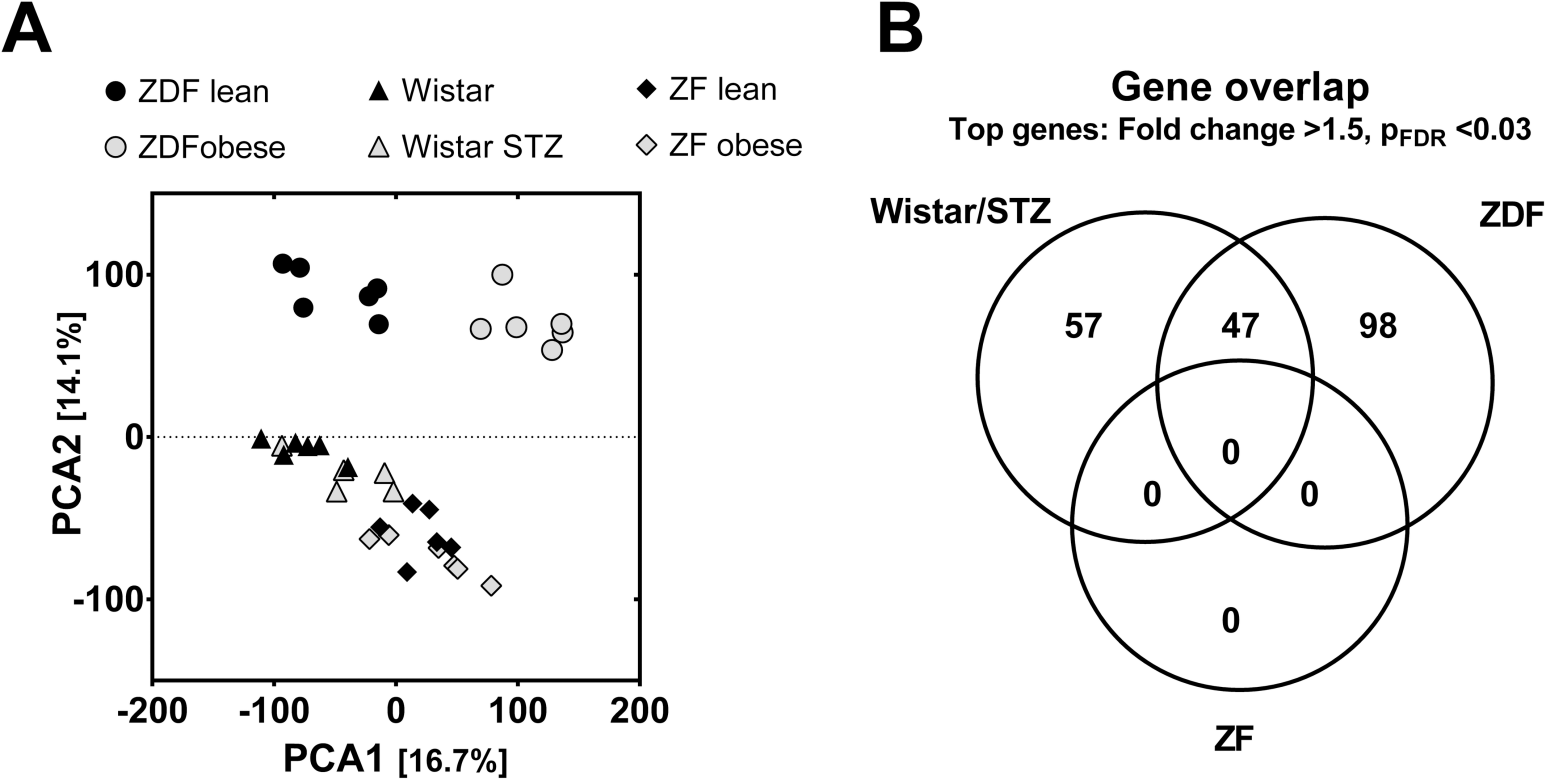
Gene array expression in retina. **A**, Principal Component Analysis on raw expression data showing the first two principal components; **B**, Venn diagram of overlap in regulated gene number between the three different animal models.

Gene expression profiling by whole transcriptome analysis using Affymetrix chip technology was used to detect differences in pathways and regulations reflecting the different modalities of the animal models. Among the probe sets on the selected chip, approximately 22,000 of 30,000 sets reflected currently known genes. Setting a minimum hybridisation level to a signal of 64 arbitrary fluorescence units, expression of 16,480 genes were analysed further by a principal component analysis to determine outliers and identify clusters (Fig 2A). Expression over all three major groups, ZDF, ZF and Wistar rats clustered already in the first two principal main components. Within the models, the most striking difference was visible between obese and lean ZDF rats. In contrast, no different clustering was observed for obese versus lean ZF rats. Setting of regulation to a minimum >1.5 fold change, revealed an major overlap on significantly regulated genes (FDR_BH<0.03) between T2D- like obese ZDF rats and T1D-like STZ-treated Wistar rats hyperglycemia (Fig 2B Venn diagram). No significantly regulated genes were identified for comparing obese versus lean ZF rat retina.

In order to confirm gene regulation assessed by micro arrays, we performed with a subset of interesting genes a confirmatory real-time PCR step. Therefore, we selected from the initial micro array 49 genes differentially regulated between ZDF obese and lean animals. These were assessed with 3 reference genes in microfluidic card PCR technology. Relative gene expression changes were calculated after normalization to the reference genes (Table 2).

**Table 2:** Comparison of microarray gene expression with confirmatory realtime PCR. From the initial micro array 49 differentially regulated genes were selected for further confirmation by real-time PCR and normalized in the PCR to the mean of three reference genes (18s, actb, gapdh). Genes were initially selected to cover a full range from highly up-regulated (positive fc values) to down-regulated (negative fc values) genes observed between ZDF obese and lean rats. Shown are fold changes (fc) and probabilities corrected for multiple testing by a Benjamini-Hochberg false discovery rate (FDR) methodology (p(FDR)).

Gene ID	ZDF obese versus lean rats				STZ treated versus non-treated Wistar rats			
	Micro array		PCR		Micro array		PCR	
	fc	p(FDR)	fc	p(FDR)	fc	p(FDR)	fc	p(FDR)
**Serpina3n**	5,4	0,01	11,76	0,09	15,2	0,0	79,4	0,02
**Gfap**	3,9	0,00	4,79	0,11	6,3	0,0	11,9	0,02
**Fgf2**	2,1	0,03	1,97	0,26	4,2	0,0	6,6	0,02
**Lad1**	3,3	0,00	3,61	0,08	3,4	0,0	4,8	0,02
**Ifi47**	3	0,01	5,03	0,08	3,3	0,0	4,1	0,13
**Serping1**	3,3	0,00	4,55	0,08	3	0,0	4,4	0,04
**C3**	2,3	0,01	3,08	0,11	2,6	0,0	3,7	0,02
**Chi3l1**	2,6	0,00	3,2	0,11	2,5	0,0	3,5	0,02
**Cp**	2,3	0,00	2,96	0,09	2,4	0,0	3	0,02
**Mx2**	2,1	0,01	3,58	0,1	2,3	0,0	11,2	0,02
**Stat1**	2,2	0,00	2,71	0,08	2,2	0,0	2,7	0,02
**Lcn2**	1,5	0,15	2,6	0,09	2,2	0,0	2,5	0,17
**Osmr**	1,8	0,01	2,2	0,14	2,2	0,0	3,6	0,02
**Oasl2**	1,7	0,01	2,8	0,42	2,1	0,0	1,1	0,49
**C1s**	2,2	0,00	3,44	0,08	2	0,0	2,6	0,15
**Stat3**	1,6	0,01	1,6	0,26	1,9	0,0	2,3	0,02
**Oas1a**	2	0,01	3,86	0,12	1,8	0,1	4,6	0,11
**Postn**	1,5	0,00	1,4	0,16	1,7	0,0	1,6	0,08
**Tnfrsf1a**	1,7	0,00	2,1	0,09	1,6	0,0	1,9	0,04
**Crym**	1,5	0,01	2	0,08	1,6	0,0	1,7	0,04
**Fcgr2b**	2	0,00	3,3	0,07	1,5	0,1	3,2	0,02
**Cxcl13**	3,2	0,00	3,99	0,08	1,5	0,4	1,3	0,48
**Slc44a2**	1,9	0,00	2,3	0,03	1,5	0,0	1,2	0,54
**C1qb**	1,6	0,01	1,1	0,55	1,5	0,1	1,1	0,49
**C1qa**	2	0,00	3,23	0,07	1,4	0,2	1,9	0,07
**C1r**	1,8	0,00	2	0,12	1,4	0,2	1,6	0,19
**Pgf**	1,3	0,03	5,1	0,03	1,3	0,2	1,6	0,33
**Sord**	1,3	0,00	1,4	0,08	1,2	0,1	1,1	0,47
**Stat2**	1,3	0,00	1,3	0,27	1,2	0,1	1,2	0,18
**Sod2**	1,2	0,00	1,3	0,09	1,1	0,2	1,1	0,25
**Tgfb2**	1,1	0,03	1,2	0,23	1,1	0,3	1	0,9
**Itgb2**	1,2	0,03	1,9	0,09	1,1	0,6	1,6	0,27
**Prkcg**	1,2	0,01	1,2	0,36	1	0,7	1,2	0,29
**Fn1**	1,3	0,00	1,4	0,26	1	0,8	-1,1	0,22
**Ager**	-1,1	0,05	-1,6	0,07	1	0,8	1,1	0,43
**Edn1**	1,1	0,04	1,3	0,12	1	0,9	1	0,97
**Ccl2**	1,5	0,04	4,6	0,09	-1	1,0	-2,4	0,62
**Rock2**	1,2	0,00	1	0,87	-1	0,9	-1	0,95
**Akt3**	1,2	0,00	1,1	0,51	-1	0,9	-1,2	0,2
**Parp1**	-1,1	0,01	-1,4	0,02	-1	0,5	1	0,9
**TNF**	-1,1	0,34	2,7	0,08	-1	0,6	1,1	0,84
**Serpinb1a**	1,2	0,04	2,1	0,03	-1,1	0,7	1,1	0,54
**Apln**	-1,1	0,02	-1	0,96	-1,1	0,3	-1,5	0,06
**Pparg**	-1,1	0,00	-1,1	0,72	-1,1	0,1	-1,3	0,42
**Igf1**	1,1	0,06	-1,2	0,11	-1,1	0,0	-1,2	0,13
**Cybb**	1,4	0,01	2,4	0,09	-1,2	0,4	-1,9	0,23
**Vegfa**	-1,1	0,10	-1,5	0,02	-1,2	0,0	-1,3	0,18
**Arr3**	-1,7	0,00	-2,4	0	-1,3	0,1	-1,9	0,12
**Lpcat1**	-1,4	0,00	-1,8	0	-1,4	0,0	-2,1	0,02

**Fig 3:**
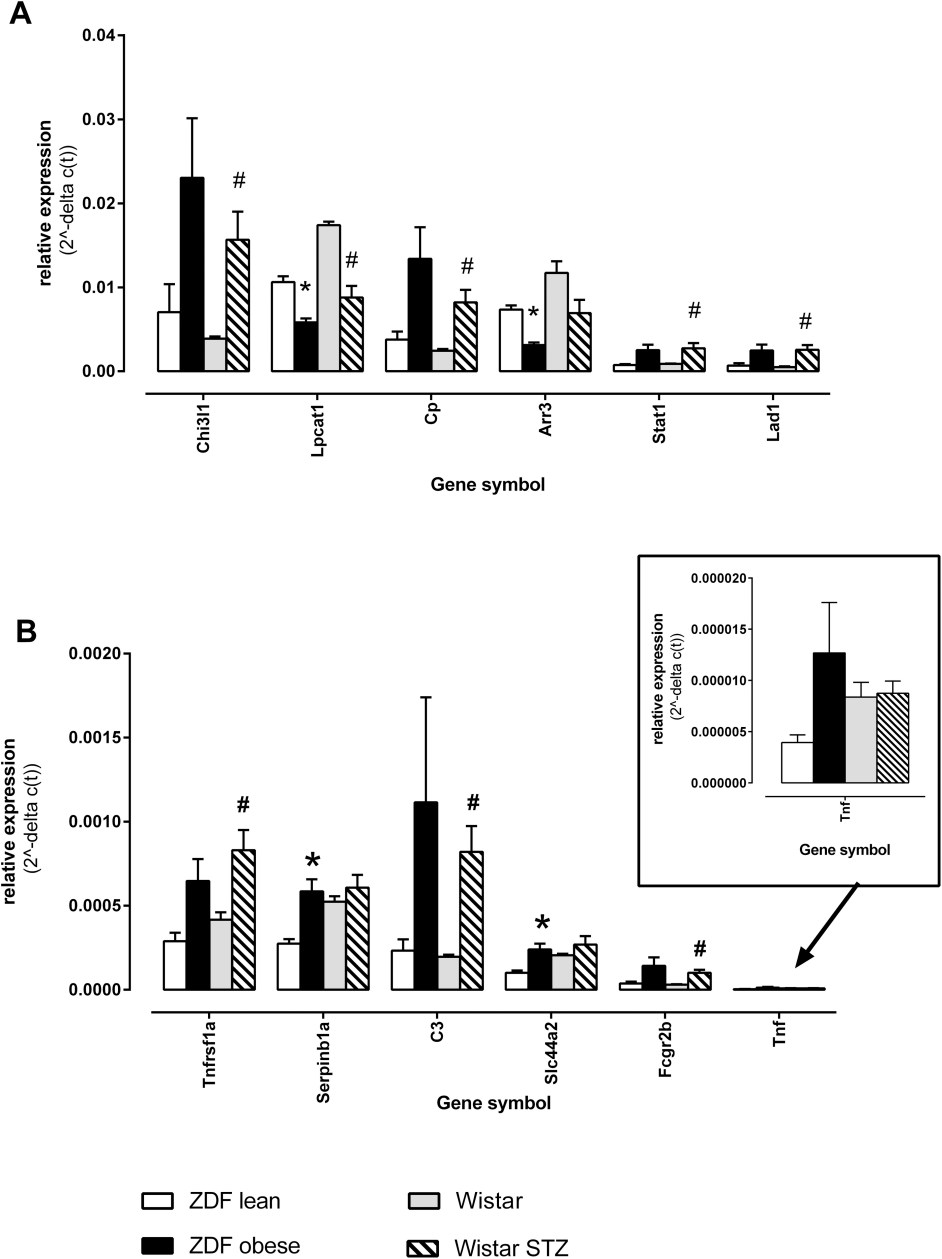
Confirmation of microarray detected gene array expression by microfluidic card PCR. **A,** relative highly expressed genes, significantly regulated in retina of ZDF obese versus ZDF lean rats; **B,** moderately and low expressed genes, significantly regulated in retina of ZDF obese versus ZDF lean rats. In the insert of Fig 3B the expression values for tnf are magnified. Shown are mean values of the relative expression and standard deviation of the mean. From each group 6 biological samples were measured. ^*)^ indicated a corrected p(FDR) value <0.05 comparing ZDF obese to ZDF lean animals; ^#)^ indicated a corrected p(FDR) value <0.05 comparing STZ treated versus non-treated Wistar rats.

The direction of expression changes initial measured by microarray could be confirmed with the PCR for all selected 49 microarray genes. For some genes like Serpina3n the PCR assessment resulted in a higher fold change. It general seemed that PCR was more sensitive in detecting changes in expression levels shown as fold changes (fc), however the micro array data often displayed lower p-values corrected for multiple testing, p(FDR). For selected genes PCR quantifications are shown in Fig 3. The expression modulation of high (Fig 3A) and medium-to low expressed genes (Fig 3B) was similar between ZDF obese versus lean rats and STZ-diabetic versus non-diabetic Wistar rats. For some genes like C3 (Fig 3B), the fold up-regulation was comparable between STZ-treated rats and ZDF obese rats but significant only in STZ-treated rats. That may be due to a too small number of replicates taken into our PCR analysis in view of the observed effect size. The inserts in Fig 3B display expression levels of tumour necrosis factor (tnf).

### Pathway analysis and upstream analysis

To identify pathways affected by both hyperglycemia, and hyperinsulinemia, we analysed mapping of genes to known pathways as defined by pathways in either Ingenuity (IPA) or MetaCore Interaction databases. A selection of the most highly represented pathways for the comparison in the T2DM and T1DM model are shown in Table 3. Pathways are shown regarding their ranking for obese versus lean ZDF rats.

**Table 3:** Overlap on differentially regulated retinal genes and top canonical pathways as defined by pathways in either Qiagen Ingenuity (IPA) or MetaCore. Pathways are arranged according to the rank for obese versus lean ZDF rats. The probability for a difference between ZDF obese versus lean and Wistar-STZ versus Wistar respectively, p(FDR), is corrected for multiple testing by a Benjamini-Hochberg false discovery rate (FDR) methodology and shown here is negative common logarithm, -lg p(FDR).

Canonical Pathways	Source	ZDF obese vs lean		Wistar-STZ vs Wistar	
		-lg p(FDR)	Rank	-lg p(FDR)	Rank
**Immune response_Classical complement pathway**	MetaCore	7,47	1	7,47	1
**Interferon Signaling**	IPA	6,71	2	1,72	32
**Complement System**	IPA	6,71	3	1,72	33
**Acute Phase Response Signaling**	IPA	6,52	4	6,08	3
**Immune response_Lectin induced complement pathway**	MetaCore	6,31	5	6,31	2
**Immune response_Oncostatin M signaling via JAK-Stat in mouse cells**	MetaCore	5,72	6	5,72	4
**Immune response_Oncostatin M signaling via JAK-Stat in human cells**	MetaCore	5,59	7	5,59	5
**Immune response_Alternative complement pathway**	MetaCore	4,95	8	4,95	6
**Oncostatin M Signaling**	IPA	4,04	9	2,82	10
**Role of Pattern Recognition Receptors in Recognition of Bacteria and Viruses**	MetaCore	3,14	10	0,26	208
**Alternative complement cascade disruption in age-related macular degeneration**	MetaCore	3,10	11	3,10	7
**Glutathione-mediated Detoxification**	IPA	3,02	12	0,48	121
**Immune response_IL-6-induced acute-phase response in hepatocytes**	MetaCore	2,96	13	2,96	8
**Attenuation of IFN type I signaling in melanoma cells**	MetaCore	2,96	14	2,96	9
**TREM1 Signaling**	IPA	2,57	15	0,31	161
**Dendritic Cell Maturation**	MetaCore	2,44	16	0,37	143
**Death Receptor Signaling**	IPA	2,26	17		
**Stem cells_Dopamine-induced expression of CNTF in adult neurogenesis**	IPA	2,25	18	2,25	11
**Development_Thrombopoetin signaling via JAK-STAT pathway**	IPA	2,25	19	2,25	12
**UVA-Induced MAPK Signaling**		2,10	20		
**Role of JAK family kinases in IL-6-type Cytokine Signaling**	IPA	2,03	21	1,93	16
**Role of IFN-beta in the improvement of blood-brain barrier integrity in multiple sclerosis**	IPA	2,02	22	2,02	13
**Release of pro-inflammatory mediators and elastolytic enzymes by alveolar macrophages in COPD**	MetaCore	2,01	23	2,01	14
**Cigarette smoke-mediated attenuation of antibacterial and antivirus immune response**	IPA	2,00	24	2,00	15
**CNTF Signaling**	IPA	1,98	25	1,22	55

**Fig 4:**
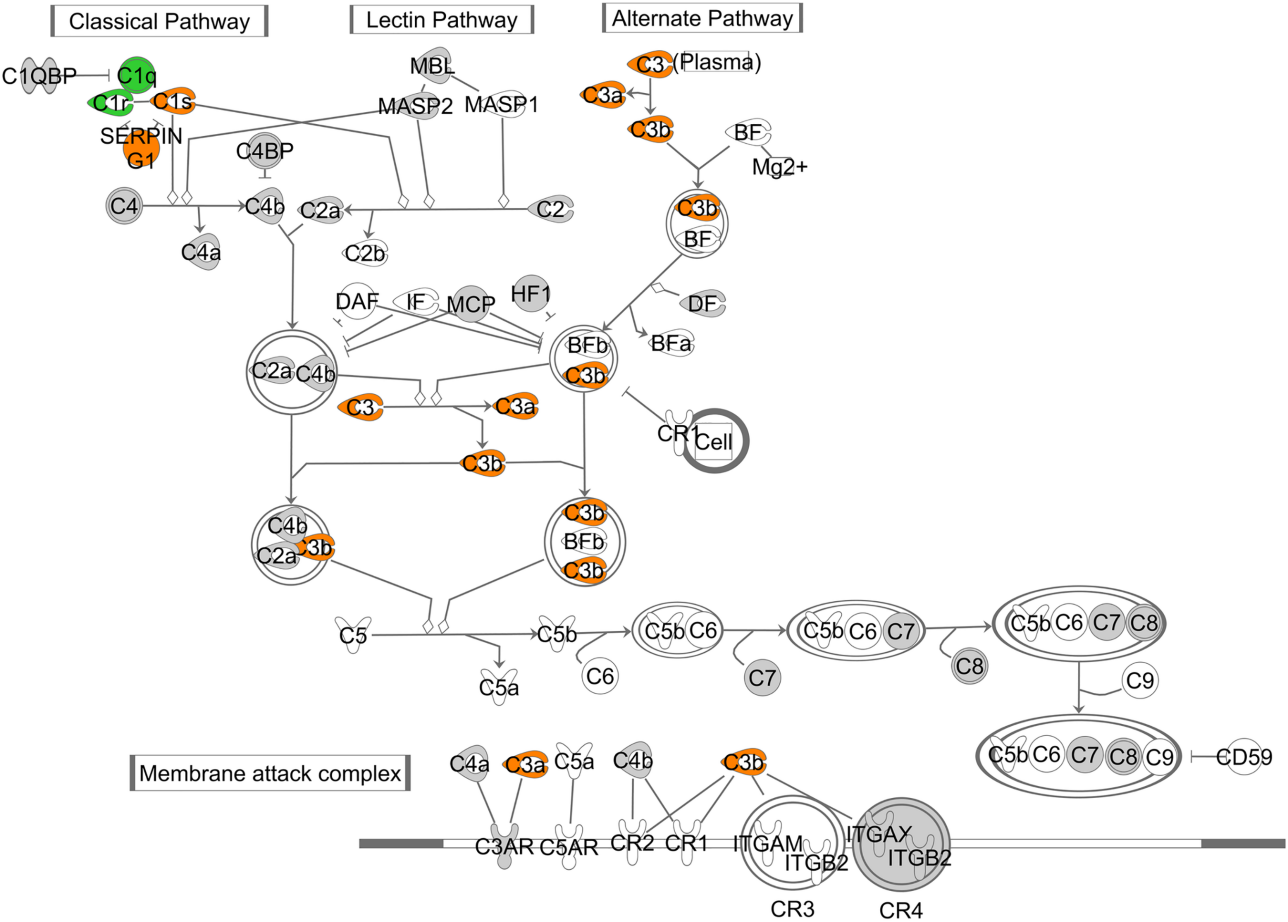
Up-regulation of the complement system in ZDF obese versus lean rats and STZ treated versus non-treated rats. Genes shown in orange color were regulated in both animal models, ZDF obese and STZ-treated rats; C1q and C1r were found regulated in ZDF but not in STZ (green color); a gray background color indicates genes which expression in retina could be confirmed but which were not regulated. Gene symbols with a transparent background color could not be assessed in the micro array measurements.

In both set of conditions, pathways reflecting activation of the innate immunity system and the complement system in the retina were observed. Regarding the complement system not all factors in this signaling cascade were detected by microarray (Fig 4, a grey color indicate expressed, a blank indicated non-expressed genes). However, among the expressed factors, a strong overlap could be detected for differential gene regulation when comparing ZDF rats to STZ rats (orange colors). Two factors in green were up-regulated in ZDF obese rats significantly. In addition to such prior broader known pathways, pathways like Oncostatin-M signaling, previously described to a limited degree in pre-clinical models of retinopathy, comprised a significant numbers of the differentially regulated genes. In analogy to the numbers of differentially expressed genes, pathway population and rank orders differed between T2DM and T1DM model.

In order to identify potential regulators which could explain the observed patterns, an upstream regulator analysis was performed with the initial gene expression micro array data. Such an analysis is based on prior knowledge of expected effects between transcriptional regulators and their target genes. In essence, it is analysed how many known targets of each regulator can be identified in the datasets. Furthermore, the direction of change, either up- or down-regulation, is compared to a prediction obtained from published data. In Table 4 the prediction of top upstream regulators for the observed differentially gene expression in ZDF and STZ models are listed. We identified α-2 and γ-Interferon, TNF-α, IL-6 and beta-Interleukin-1 as transcriptional regulators of inflammation. Most notably, almost all predicted transcription upstream factors were not found to be differentially regulated on gene expression level.

**Table 4:** Top upstream regulators predict to explain the gene expression differences in retina between obese and lean ZDF rats or STZ treatet and non-teated Wistar rats. Molecules are sorted by the modulation z-score in ZDF rats.

Upstream regulator		ZDF obese vs lean				Wistar-STZ vs Wistar			
Gene name	Hugo Gene Identifier	Predicted modulation state	modulation z-score	p-value of modulation	Fold change in obese versus lean ZDF rats	Predicted modulation state	modulation z-score	p-value of modulation	Fold change in STZ treated vs non-treated rats
**Interferon, gamma**	**IFNG**	Activated	4,53	2,90E-27	nd	Activated	3,61	8,80E-15	nd
**Interferon, alpha 2**	**IFNA2**	Activated	3,84	7,50E-21	nd	Activated	2,045	3,17E-06	nd
**Interleukin 1, beta**	**IL1B**	Activated	3,35	3,90E-20	1,01		1,72	2,90E-13	-1,04
**Oncostatin M**	**OSM**	Activated	4,17	1,70E-19	-1,18	Activated	3,39	3,90E-11	-1,07
**Signal transducer and activator of transcription 1**	**STAT1**	Activated	2,86	1,80E-19	2,19	Activated	2,14	2,60E-09	2,24
**Interleukin 6**	**IL6**	Activated	4,02	1,70E-18	nd	Activated	3,04	4,80E-13	
**Tumor necrosis factor, alpha**	**TNF**	Activated	4,06	2,00E-16	-1,04		1,84	1,00E-09	-1,09
**Tuberous sclerosis 2**	**TSC2**	Inhibited	-3,61	2,10E-14	-1,15		-1,63	6,36E-06	-1,086
**Signal transducer and activator of transcription 3**	**STAT3**	Activated	2,72	2,50E-13	1,58	Activated	2,48	1,70E-07	1,91
**Janus kinase 2**	**JAK2**	Activated	3,09	1,60E-12	1,08	Activated	2,62	3,50E-08	1,15
**Mitogen-activated protein kinase 1**	**MAPK1**	Inhibited	-3,85	2,7E-11	-1,05		-0,97	1,40E-07	-1,01
**SWI/SNF related, matrix associated, actin dependent regulator of chromatin, subfamily a, member 4**	**SMARCA4**	Activated	2,77	2,14E-04	1,035		-0,28	1,20E-08	-1,05

**Fig 5:**
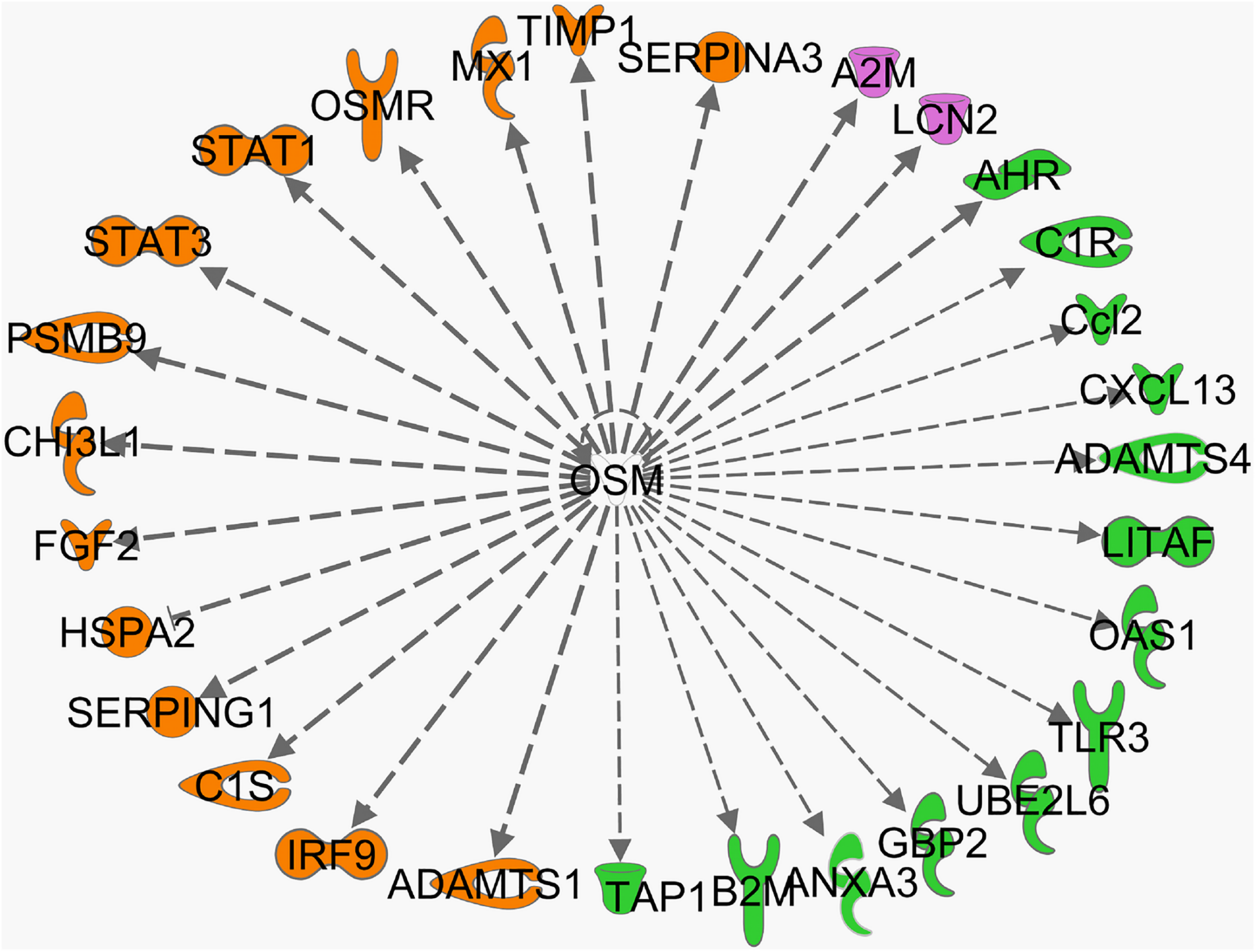
Relationship between oncostatin M (OSM) and literature known downstream effector genes in microarray data sets. Genes highlighted in orange were regulated in obese versus lean ZDF rats and STZ treated and non-treated rat retina. Genes highlighted in green were regulated in obese versus lean ZDF but not in STZ treated vs non-treated Wistar rat retina. Genes highlighted in purple were differentially regulated in STZ treated versus non-treated rats but not in obese versus lean ZDF rat retina.

Together, our analysis added new upstream regulators such as oncostatin-M to glycemia-dependent regulators of gene expression changes in experimental retinopathy models. The relationship between oncostatin M and literature known downstream effector genes is graphically represented for our micro array data sets (Fig 5). A high number of oncostatin-M modulated genes were regulated in obese versus lean ZDF rats and STZ treated and non-treated rat retina; among them were stat1 and stat3. A number of genes were regulated in obese versus lean ZDF but not in STZ treated vs non-treated Wistar rat retina. Only two oncostatin-M downstream genes, lcn2 and a2m, were differentially regulated in STZ treated versus non-treated rats but not in obese versus lean ZDF rat retina. Overall 29 downstream genes are shown; for a subset of 11 selected genes expression changes were assessed by real-time PCR including lcn2, c1r, ccl2, oas1, c1s, serping1, fgf2, chI3l1, stat1, stat3, and osmr.

## Discussion

In this study, we analysed retinal gene expression patterns and compared three conditions, which are thought to contribute to the incipience of diabetic retinopathy, i.e. hyperglycemia, hyperinsulinemia, and the combination of both. Interestingly, we found that hyperinsulinemia alone as assessed in ZF rats did not change gene expression in the retina despite substantial metabolic alterations usually implied in retinal vascular pathology. In sharp contrast, a substantial overlap in gene regulation was found in the two phenotypically opposite models which share hyperglycemic, but not insulin levels.

There is ample evidence that both, STZ and ZDF rats develop features of preclinical, early diabetic retinopathy [6] [11]. STZ treated rodents are often named as T1D model, despite the lack of any auto-immune responses [12]. However, in this model many drivers of late complications in T1DM patients are fully established such as hypoinsulinemia with ketone body elevation and hyperglycemia). In contrast to STZ and ZDF rats, ZF rats have very mild retinal pathology only at old age and in combination with glucose intolerance but not at younger age [13]. Thus, as our first important notion, the metabolic changes in obese, non-hyperglycemic but hyperinsulinemic ZF rats do not cause strong alterations in retinal gene expression and retinal (vascular) pathology. In this context, it should be noted that our data comply with previous notions that hyperinsulinemia in combination with lipid abnormalities in ZF rats is insufficient to alter retinal vascular modifiers such as VEGF despite increased systemic oxidative and inflammatory stress markers [14].

Our second important observation is the identification of the metabolic patterning of the two models which coincide in retinal pathology. Beyond the plasma concentrations of insulins, obese ZDF and STZ-diabetic (lean) rats differ substantially in some metabolic parameters. We found strongly elevated ketone bodies and free fatty acids to be elevated in obese ZDF rats but not in STZ-diabetic rats. Clinically, increased ketone body formation is associated with neurotoxicity, in particular in type 1 diabetes. In contrast, acetoacetate and ß-hydroxybuyrate protect from N-methyl-D-aspartate induced damage of retinal ganglion cells in rats [15]. Another difference between models is the level of hyperlipidaemia, observed in obese ZDF rats but not in STZ-treated rats. Kowluru and colleagues recently considered dyslipidemia in the hyperglycemic milieu as one of the major factor in the development of vascular changes in retinopathy [16]. This is in contrast with our experimental data, which suggest that, despite high levels of FFA, triglycerides and cholesterol, gene expression levels remain completely unchanged. Retinopathy in patients with good control of low-density lipoprotein cholesterol, were less robustly associated with plasma triglycerides and lower levels of high-density lipoprotein cholesterol supporting more a weak influence of dyslipidemia to retinal disease progression [17]. In human patients, diabetic retinopathy pathologies occur only to a low degree in patients who only have hyperlipidemia and insulin resistance without hyperglycemia or diabetes. As a clinical correlate to our pre-clinical ZF rat findings, it has been recently described that severely obese patients with strong dys- and hyperlipidemia have only a low grade of retinopathy assessed as microaneurysms or dot hemorrhages, while diabetes increases the risk for retinopathy by 8.3 fold[18].

Our third and most important notion revealed, that STZ-induced diabetic and ZDF rats share a strong overlap pathways analysis using known interaction databases like Meta-Core and Ingenuity, e.g. in the activation of the complement system. Previous data had linked the complement system to different diabetic angiopathies like diabetic kidney in particular[19]. As an immune privileged tissue, the retina has a unique immune defence systems consisting of microglia and the complement system. The complement system sub-divides into three different activating pathways and consists of over 30 proteins. These factors are involved in maintenance of cellular homeostasis, protection from cell stress and death, and removal of cell debris. Under physiological conditions, a low-grade activation of the complement system may be important for retinal homeostasis. Dysregulation and stronger activation in retinal pathologies may then result in detrimental chronic inflammation. [20]. Our microarray data are in accordance with earlier data, that the retina only expresses selected components of complement system, although we cannot rule out technical issues in microarray probe technology, false negatively indicating absence of certain components. In particular, the formation of the membrane attack complex (MAC) and the regulation of CD 59, known to be modulated by hyperglycemia and AGE formation, was not observed, but needs further specification, since this is crucial for a more complete understanding. In future studies we would suggest to probe with a sensitive and reliable technology like the microfluidic PCR technology, all possible components of the complements in parallel. In addition to such basic studies, further work need to be envisaged, how the complement system is activated in the diabetic eye [20]. We observed in our pre-clinical models an up-regulation of the C3 convertase, and in addition of C1q and C1r in the ZDF rat retina. However, the notion that not only complement activation, but also inhibitors of activation such as SERPING3 were strongly up-regulated on our models, needs further consideration, since complement targeted therapy is currently a high active research and development area [21].

It is surprising from our gene array analysis, that changes such as VEGF found to be increased in diabetic patients were not found to be elevated in our rodent models. Most of the relevant human studies on VEGF expression have been assessed with retina in final stages of diabetic retinopathy, e.g. proliferative diabetic retinopathy. Despite many attempts to establish suitable models that reflect the features of such late human disease stages, our selected animal models do not develop such severe disease stages with large areas of retinal non-perfusion and neovascularization, in accordance with other rodent models (for a review see [12]). As we confirmed our expression findings in general by two independent technologies, we would like to rule out shortcomings in methodology to assess gene expression, with one exception, in that we assessed with both methods total mRNA for the whole tissue. Hence, up-regulation localized to certain layers of the retina may not have been detected sufficiently strong enough.

In previous study, we detected only minor changes in key inflammatory genes in retina from ZDF rats assessed by microfluidic PCR directly and concluded that inflammation may only be a minor mechanism in experimental diabetic retinopathy [6]. Our new micro array data first confirm the expression finding. TNF-α mRNA is expressed only on a very low level in obese ZDF rat retina and not even regulated in STZ-treated rats. However, after performing now pathway analysis and the identification of upstream (transcription) activators, we need to add that TNF-α, α- and γ-interferons and beta-interleukin-1 are among the top upstream regulators which can explain most of the many downstream gene expression changes in both diabetes models. We speculate that this seemingly discrepancy can be explained either by a predominant regulation on a post-transcriptional rather than a transcriptional level or, by regulatory changes at earlier time points and visible in our selected later time point only by the secondary expression changes of downstream genes.

Among the top upstream activators and pathways is oncostatin-M (OSM). OSM is a member of the gp130 family of cytokines with pleiotropic functions and is a secreted cytokine involved in chronic inflammation [22]. It was first discussed in cancer in the context of inhibiting the proliferation of melanoma cell lines. In contrast to other gp130 cytokines, OSM may have particular functions because of unique pathways downstream of the OSM receptor (OSMR), which is formed as a heterodimer of OSM-receptor-β and gp130. A strong overlap in oncostatin-M downstream genes between ZDF obese and STZ rat retina could be observed, among them were two isoforms of signal transducer and activator of transcription genes, stat1 and stat3. Stat1 is already known to be involved in apoptosis of retinal pericytes under high glucose conditions [23]. Recent findings identified stat3 as regulator in survival and inflammatory response of the retinal pigment epithelium (RPE)[24].

To date, a few studies investigated the role of OSM and its receptor in the retina. Rattner *et al* could demonstrate, that the OSMR is expressed in RPE, and one of the few transcripts strongly up-regulated in a murine model of retinal light damage, putatively reflecting a stress signal response [25]. Simo and colleagues collected evidences that a breakdown and dysfunction of RPE may have an important role in diabetic retinopathy (for a review see [26]. Using immune-cytochemistry with an antibody directed against OSMR, we would suggest to investigate whether the up-regulation of OSMR observed in our diabetes models for lysates taken from the whole retina, is localized to the RPE layer within the retina. Recombinant OSM, protected both rod and cone photoreceptors from degeneration in a transgenic rat model of retinal degeneration [27]. In further support of the concept that the OSM/OSMR systems serves a pro-survival neurotrophic function, a single injection of recombinant OSM, in a mouse model of optic nerve injury, improved retinal ganglion cell survival and function [28]. More direct data on the function of OSM in the diabetic retina may result from studies in OSMR deficient mice. Such mice are fertile and mostly healthy [29]. In our studies, the OSMR was found consistently up-regulated in ZDF obese rats and STZ-treated rats compared to respective controls. As a first next step, we would suggest to treat OSMR deficient mice with STZ and measure vasoregression as relevant early time point in retinopathy.

In conclusion, we observed systemic hyperglycemia contributing strongly to the initiation and progression of experimental diabetic retinopathy, whereas isolated systemic hyperinsulinemia fails to induce significant gene regulation, even in association with other metabolic abnormalities. Our unbiased microarray gene expression analysis revealed a strong overlap between T1D and T2D models of late complications, with similar known but also new pathways modulated under both disease conditions. Upstream analysis turned out to detect key underlying transcription factors which were by itself mostly not regulated on a gene expression level.

## Supporting information

S1 Table Primers and c(q) PCR data for confirmatory PCR. Genes, assay ID and c(q) data and corresponding sample list.
(XLS)
